# The characteristics and trend of adverse events following immunization reported by information system in Jiangsu province, China, 2015–2018

**DOI:** 10.1186/s12889-021-11387-3

**Published:** 2021-07-07

**Authors:** Ran Hu, Shanshan Peng, Yuanbao Liu, Fengyang Tang, Zhiguo Wang, Lei Zhang, Jun Gao, Hongxiong Guo

**Affiliations:** 1 Jiangsu Provincial Center for Disease Control and Prevention, Nanjing, China; 2 Department of Hepatology, The Second Hospital of Nanjing, Nanjing University of Chinese Medicine, Nanjing, China

**Keywords:** Adverse events following immunization, Incidence of adverse events following immunization, Serious vaccine reaction, Common vaccine reaction, Vaccine, Categories of vaccine reaction

## Abstract

**Objective:**

Adverse events following immunization is an important factor influencing public trust in vaccination. Publicizing its incidence timely can increase public trust. The aim of this study is to describe the incidence and characteristics of adverse events following immunization in Jiangsu province of China from 2015 to 2018.

**Methods:**

All information of adverse events following immunization (AEFIs) was gained from Jiangsu Province Vaccination Integrated Service Management Information System. The reported AEFI trend was analyzed using Chi-square test.

**Results:**

A total of 77,980 AEFI cases were reported through the AEFI system; Among which, 77,731 were classified as non-serious AEFI cases and 249 were serious AEFI cases. The male to female ratio was 1.31:1, cases less than 7 years old accounted for 97.7%. The total estimated AEFI rate was 62.70/100,000 doses. By severity, 60.75/100,000, 4.46/100,000 and 0.11/100,000 AEFI cases were common vaccine reaction, rare vaccine reaction, and serious rare vaccine reaction, respectively. The top two serious AEFI were thrombocytopenic purpura and febrile. The incidence rates showed the increasing trend and the linear trend of the increasing incidence rates passed the significant test at 0.05 levels.

**Conclusion:**

The sensitivity of AEFI monitoring in Jiangsu Province is increasing and higher than the national average and most countries. The majority of AEFI cases were common adverse reactions, while the serious vaccine reactions caused by vaccines were extremely low. To elevate the sensitivity of AEFI surveillance may reduce the incidence of developing serious AEFI cases.

## Background

Immunization is the most effective and cost-effective means of public health intervention [1, 2]. Vaccine do not only prevent vaccinated population from getting agents infection and developing a potentially serious illness, but also protect entire communities by reducing their spread [3, 4]. It has led to the global eradication of smallpox as well as the elimination of poliomyelitis in regions of the world [5]. Due to the low prevalence and incidence of vaccine-preventable diseases, the public attention on adverse events following immunization (AEFI) has being increased in recent years [6]. Especially vaccine hesitancy, which often becomes anti-vaccine movement in some regions, even lead a resurgence of some vaccine-preventable diseases [6, 7].

To maintain a good public trust, many countries established surveillance system on vaccine safety. In mainland of China, a passive AEFI Information System (AEFIIS) was established in 2008 [8, 9], and updated twice in 2015, 2018, respectively. The parent of child often reports the symptoms of their child occurred after vaccination to physicians at vaccination clinic, then physicians report this event to AEFIIS if they confirm that it meets the criteria of AEFI. In addition, when clinicians find these AEFIs, they will contact the public health doctors at vaccination clinic. Public health doctors will report them to AEFIIS. All AEFIs monitoring reports and diagnostic requirements are based on National Regulatory Authority evaluation requirements, which is consistent with WHO requirements. However, the AEFIIS is not enough to meet the requirement from various provinces in China. They built their own information system according to their own requirements, and more specific indications are included in provincial ones compared to the Nation’s. In this system, all reported AEFIs are collected. Nonetheless, little data about the characteristics of AEFI from this system was reported.

Jiangsu province, located in the east of China, is a developed region with an approximate 80 million people. Every year, more than 20 million doses were vaccinated. Jiangsu province has been committed to developing its own vaccine information system (Jiangsu Province Vaccination Integrated Service Management Information System) [10]. In this system, the information included the traceability and batch number of vaccine and ones of all vaccinees were collected. Every year, more than ten thousand AEFIs were documented since 2015. By using the AEFI monitor system and the traceability system, we may obtain more accurate vaccination information in case of AEFI. In this study, we reported the basic characteristics of reported AEFI from Jiangsu provincial AEFIs.

## Materials and methods

### AEFI data

AEFI case data and vaccination information were gained from Jiangsu Provincial AEFI information management system, which covered all AEFI cases collected from January 1, 2015 to December 31, 2018 and information of 53 vaccines.

### The definition of AEFI

AEFIs were classified into adverse event following immunization, vaccine quality, program error, coincidental event and psychogenic reaction. Vaccination-related reactions or events include fever (axillary temperature ≥ 38.6 °C), local redness (diameter > 2.5 cm), local induration (diameter > 2.5 cm), allergic skin rash (including urticaria, maculopapular rash, measles, scarlet fever-like rash), angioedema, anaphylactic shock, allergic laryngeal edema, Henoch schonlein purpura (HSP), thrombocytopenic purpura (TP), local allergic necrosis (Arthus response), febrile seizures, epilepsy, brachial plexus neuritis, polyneuritis, Guillain-Barré syndrome (GBS), Acute disseminated encephalo-myelitis (ADEM), encephalitis and meningitis, encephalopathy, Vaccine-associated paralytic poliomyelitis (VAPP), BCG lymph nodes Inflammation, BCG osteomyelitis, disseminated BCG infection, aseptic abscess, local abscess, lymphangitis and lymphadenitis, cellulitis, systemic purulent infection (poison) Disease, sepsis, sepsis), toxic shock syndrome, syncope, hysteria and suspicion and vaccinations related to other serious AEFI. Severe AEFI is an AEFI that causes death, life-threatening, permanent or significant disability or organ function damage, including allergic laryngeal edema, anaphylactic shock, HSP, TP, arthus response, febrile seizures, epilepsy, brachial plexus neuritis, polyneuritis, GBS, ADEM, encephalopathy, encephalitis and meningitis, VAPP, BCG osteomyelitis, disseminated BCG infection, syncope, toxic shock syndrome, generalized purulent infection [11].

### Statistics analysis

The data of AEFI cases in China’s AEFI information management system from 2015 to 2018 were exported to Microsoft Excel for statistical analysis. If two or more vaccines are vaccinated at the same time, the most suspected vaccine will be included in the calculation. Trend test was calculated by χ^2^ with R. In this study, the north area includes Xuzhou, Huai’an, Lianyungang, Suqian, Yancheng, Nantong, Yangzhou, Taizhou; The south area includes Nanjing, Zhenjiang, Changzhou, Wuxi, Suzhou. The incidence rate of an AEFI for a vaccine (100,000 doses) = the number of AEFI reports / the number of vaccinations × 100,000 doses.

## Results

### The baseline characteristic of AEFI cases

From 2015 to 2018, a total of 77,980 AEFI cases were reported. The average incidence of AEFI is 65.4 per 100,000 doses. Of which, 77,686 were adverse reactions, accounting for 99.62% (72,377 were common vaccine reactions, while 5309 were rare vaccine reactions); 285 were coincidental events (0.37%); 8 were cases of psychogenic reaction (0.009%); 1 was program error (0.001%); no vaccine quality accident was reported. As shown in Table 1, the incidence of AEFI in male was higher than that in female. 97.7% of total AEFI was reported in children less than seven years old, especially in children younger than one year old, which took account for 56.36%. More AEFIs occur between April and September every year. Non-serious AEFI and Serious AEFI shared with the similar baseline characteristics.

**Table 1:** Baseline characteristic of AEFIs from 2015 to 2018 in Jiangsu, China

	Non-serious AEFI		Serious AEFI		Total	
Characteristic	No. of cases	Proportion	No. of cases	Proportion	No. of cases	Proportion
Gender						
Female	33,605	43.23	106	42.57	33,711	43.23
Male	44,126	56.77	143	57.43	44,269	56.77
Age		0.00				
≤ 1y	43,790	56.34	158	63.45	43,948	56.36
2y-6y	32,174	41.39	68	27.31	32,242	41.35
≥ 7y	1767	2.27	23	9.24	1790	2.30
Area						
North	41,573	53.48	102	40.96	41,675	53.44
South	36,158	46.52	147	59.04	36,305	46.56
Month						
Jan-Mar	14,924	19.20	65	26.10	14,989	19.22
Apr-Jun	25,902	33.32	64	25.70	25,966	33.30
Jul-Sep	23,683	30.47	71	28.51	23,754	30.46
Oct-Dec	13,222	17.01	49	19.68	13,271	17.02
Total	77,731	100	249	100.00	77,980	100.00

### Vaccine-specific reporting rate for vaccine adverse events

The highest incidence rates of common vaccine reactions were DTaP-IPV/Hib (251.27/100,000), PPV23 (242.66/100,000) and DTaP (198.58/100,000). The highest incidence rates of rare vaccine reactions were MR (46.76/100,000), MV (46.01/100,000) and PCV7 (24.97/100,000). The highest incidence rates of AEFI were DTaP-IPV/Hib (258.94/100,000), PPV23 (248.98/100,000) and DTaP (204.27/100,000) (as shown in Table 2).

**Table 2:** Numbers, proportions and estiamted incidence rates (per 100,000 doses) of AEFIs by vaccine in Jiangsu, China

Vaccine	Common Vaccine Reaction			Rear Vaccine Reaction			AEFI		
	No. of cases	proportion(%)	Estimated incidence	No. of cases	proportion(%)	Estimated incidence	No. of cases	proportion(%)	Estimated incidence
BCG	295	0.408	7.33	346	6.521	8.60	658	0.84	16.35
HepB (CHO)	116	0.160	11.48	14	0.264	1.39	131	0.17	12.96
HepB (YEAST)	3787	5.234	33.10	192	3.619	1.68	4017	5.15	35.11
tOPV	402	0.556	6.85	54	1.018	0.92	464	0.60	7.91
IPV (Sabin)	363	0.502	65.01	34	0.641	6.09	401	0.51	71.82
IPV (Salk)	645	0.891	25.28	70	1.319	2.74	736	0.94	28.84
bOPV	917	1.267	13.52	125	2.356	1.84	1054	1.35	15.54
DTaP	29,464	40.719	198.58	757	14.267	5.10	30,278	38.84	204.07
DT*	5905	8.161	166.41	61	1.150	1.72	5974	7.66	168.35
Tetanus	1	0.001	1.30	1	0.019	1.30	2	0.00	2.60
MV	298	0.412	126.95	108	2.035	46.01	410	0.53	174.67
MMR	1821	2.517	43.09	301	5.673	7.12	2131	2.73	50.42
MM	7	0.010	57.27	0	0.000	0.00	7	0.01	57.27
MR	4717	6.519	121.11	1821	34.320	46.76	6558	8.41	168.38
MPV-A	7811	10.795	100.49	316	5.956	4.07	8153	10.46	104.89
MPV-AC	2776	3.836	38.18	164	3.091	2.26	2952	3.79	40.60
MPCV-AC	83	0.115	32.43	6	0.113	2.34	89	0.11	34.78
MPV-ACYW135	64	0.088	38.60	3	0.057	1.81	68	0.09	41.01
JEV-L	3754	5.188	46.34	328	6.182	4.05	4089	5.25	50.48
JEV-I	80	0.111	49.35	7	0.132	4.32	87	0.11	53.67
HepA-L	1	0.001	0.42	0	0.000	0.00	1	0.00	0.42
HepA-I	2198	3.038	28.41	136	2.563	1.76	2339	3.00	30.23
HepAB	20	0.028	34.40	1	0.019	1.72	21	0.03	36.12
InfV	539	0.745	41.40	34	0.641	2.61	579	0.74	44.47
VarV	824	1.139	38.95	100	1.885	4.73	932	1.20	44.06
Hib	956	1.321	74.17	64	1.206	4.97	1020	1.31	79.13
ORV	109	0.151	27.58	7	0.132	1.77	117	0.15	29.61
PPV23	845	1.168	242.66	21	0.396	6.03	867	1.11	248.98
PCV7	23	0.032	95.73	6	0.113	24.97	29	0.04	120.70
PCV13	219	0.303	127.33	16	0.302	9.30	236	0.30	137.21
RabV	1037	1.433	5.11	76	1.432	0.37	1123	1.44	5.53
DTap-Hib	274	0.379	169.66	12	0.226	7.43	286	0.37	177.09
DTaP-IPV-Hib	1309	1.809	251.27	34	0.641	6.53	1349	1.73	258.94
HepE	6	0.008	29.19	0	0.000	0.00	6	0.01	29.19
MPV-AC/Hib	132	0.182	51.98	13	0.245	5.12	145	0.19	57.10
EV71(Human Diploid)	282	0.390	34.39	40	0.754	4.88	328	0.42	40.01
EV71(Vero)	249	0.344	64.83	31	0.584	8.07	283	0.36	73.68
B-HPV	14	0.019	9.23	3	0.057	1.98	18	0.02	11.87
Q-HPV	16	0.022	10.12	4	0.075	2.53	21	0.03	13.28
Total	72,359	100.000	60.68	5306	100.000	4.45	77,959	100.00	65.38

### Characteristics of vaccine adverse events cases and its distribution

Common reaction associated with vaccination consists of high fever, redness and swelling in shot site, and scleroma. Of them, high fever takes account for 42.73%, followed by redness and swelling in shot site (38.81%). Abnormal reaction includes allergic reaction, nervous system response, BCG specific reaction, and injection site reaction, and others. Among abnormal reaction, allergic reaction is the major, which accounts for 89.88%. Among allergic reaction, allergic skin rash is the predominant, reaches to 94.05%. Nervous system response and BCG specific reaction reaches to 2.37 and 0.52%, respectively (as shown in Table 3).

**Table 3:** The symptoms and signs of AEFIs from 2015 to 2018 in Jiangsu, China

AEFI	Total
Common reaction	
High fever	34,762
Redness and swelling	31,576
scleroma	15,013
Abnormal reaction	
Allergic reaction	
Anaphylactic shock	2
Allergic purpura	12
Edema of larynx	1
Arthus reaction	2
Angioedema	48
Thrombocytopenic purpura	49
Allergic skin rash	4060
Other allergic reactions	143
Nervous system response	
Febrile convulsion	17
Acute disseminated encephalomyelitis	2
epilepsy	2
encephalopathy	1
Vaccine associated paralytic poliomyelitis	1
Other nervous system reactions	2
BCG specific reaction	
BCG lymphadenitis	94
Systemic disseminated BCG infection	2
BCG local abscess	14
Other reactions of BCG	4
Injection site reaction	
sterile abscess	34
Other local reactions	6
Others	307
Total	86,154

People vaccinated against DTaP and DT are more likely to have induration (DTaP, 71.23/100,000; DT, 65.01/100,000), redness and swelling (DTaP, 148.88/100,000; DT, 132.83/100,000). People vaccinated against MR and MPV-A are more likely to have fever (MR,119.60/100,000; MPV-A, 84.79/100,000). People vaccinated against MR and MMR are more likely to have allergic skin rash (MR, 45.16/100,000; MMR, 6.53/100,000). BCG lymphadenitis is more common, compared to other specific reactions of BCG.

### Severity of the vaccine adverse events

As shown in Table 4, from 2015 to 2018, serious AEFI cases reaches to 249. Among them, abnormal reaction takes account for 52.61%, while psychogenic reaction takes account for 45.78%. The main abnormal reaction is allergic reaction thrombocytopenic purpura which reaches to 53, followed by febrile convulsion for 26 and Henoch Schonlein purpura for 14.

**Table 4:** The distribution of various severe AEFIs reported from 2015 to 2018 in Jiangsu, China

Final clinical diagnosis	Abnormal reaction	Coincidence	Psychogenic reaction	Total
Febrile convulsion	26	24	0	50
Allergic reaction - anaphylactic shock	8	0	0	8
Allergic reaction - Henoch Schonlein purpura	14	7	0	21
Allergic reaction thrombocytopenic purpura	53	24	0	77
Allergic reaction - laryngosis edema	2	1	0	3
Allergic reactions - other allergic reactions	0	2	0	2
Guillain-Barre Syndrome	1	1	0	2
Epilepsy	2	7	0	9
Encephalopathy	1	2	0	3
Encephalitis and meningitis	1	2	0	3
Vaccine associated paralytic poliomyelitis	3	0	0	3
Systemic BCG infection	2	0	0	2
Systemic suppurative infection - toxemia	0	1	0	1
Systemic suppurative infection sepsis	0	3	0	3
Syncope	1	1	3	5
Acute disseminated encephalomyelitis	4	1	0	5
Other	13	38	1	52
Total	131	114	4	249

### The trend of AEFI over year

**Fig. 1:**
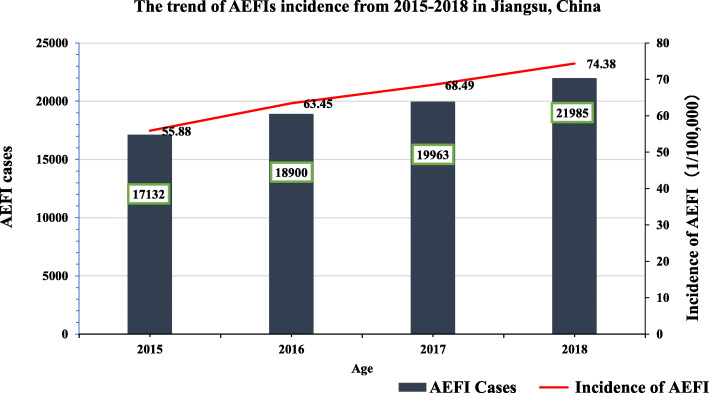
The trend of AEFIs incidence from 2015 to 2018 in Jiangsu, China

From 2015 to 2018, AEFI incidence per 100,000 doses in 2015, 2016, 2017, and 2018 was 55.88, 63.45, 68.49, and 74.38, respectively (as shown in Fig. 1). The reported AEFI incidence has being increased at a rate of nearly 9% since 2015.

## Discussion

The reported incidence of AEFI in Jiangsu during 2015–2018 was 69.70/100,000 doses, which is significantly higher than the average AEFI incidence reported in the whole China [11] and the incidence of AEFI reported in other countries [12–16]. According to the 2016 annual report released by the National Center for Immunization Planning, the reported incidence of AEFI in China was 39.01 /100,000 doses [11]. In USA, the reported incidence of AEFI reached to 11.4/100,000 doses in 1999–2001 and approximately 12.8/100,000 doses after 2013 [12]. In Australia, an annual AEFI reporting rate of 16.9/100,000 doses was administered in 2018, which was closed to the average annual AEFI reporting rate (13.4/100,000 doses) distributed in Canada for vaccines administered during 2013–2016 [15, 17].

Higher reported incidence of AEFI in Jiangsu Province of China may be contributed partially from the sensitivity of AEFI monitoring system. In Jiangsu province, all potential AEFI cases should be reported with 48 h, and the follow investigation be conducted within the second 48 h, and all questionnaires be uploaded within 72 h. All community doctors were trained periodically, and adverse reaction after vaccination were widely publicized for parents and guardians of children in clinics and society news media. All these endeavors increased the sensitivity of AEFI monitoring system. Especially since 2015, training for community doctors and publicizing the knowledge about vaccination were enforced. Moreover, the monitoring system was updated for many times. Consequently, the sensitivity of monitoring AEFI gradually rose, and the reported AEFI incidence increased at a rate of about 9% since 2015.

Among all reported AEFI cases, the incidence of the severe cases accounted for 0.32% during the four-year period, which was less than the average incidence in the whole China (0.78%, 2015–2016, 11] and those in Australia (16%, 2018, 14] and the United States (7%, 2011–2014, 12]. The difference on the incidence of severe AEFIs between countries partially was caused by the different definition of severe AEFI in various countries. However, the incidence of severe AEFIs may be reduced by other factors such as local medical resources, the measures associated with vaccination, the sensitivity of monitoring system on AEFI, even economic level. Jiangsu province, as one of the most developed provinces in China, has higher public health service, medical resource, communication system. Furthermore, all people were required to stay for another 30 min after they were vaccinated in Jiangsu province, all vaccination clinics qualify to administrate this reaction. As we knew, the immediate hypersensitivity is one of severe AEFIs [18]. Moreover, higher sensitivity on monitoring AEFI may prevent many AEFI cases from developing severe ones. More than that, 0.03% death cases of AEFIs are lower in Jiangsu province than in the whole China (0.07%, 2015–2016) and Australia (0.16%, 2014–2015). Of the 26 deaths, 24 were eventually diagnosed as coincidental. Coincidental serious diseases were the main causes of death, which was similar to the monitoring situation of death cases in the whole China and the United States vaccine adverse event reporting system (VAERS) from 1997 to 2013 [19].

The incidence and symptom of AEFIs are associated with vaccines’ complement. For example, the incidence of AEFI is higher in population vaccinated with DTaP, DT and MPV-A vaccines than others [20, 21]. MPV-A, MR and JEV-L have been reported to have a higher incidence of inducing fever [22], while DTaP, DT and other DTaP-related vaccines have been reported to have a higher incidence of causing local redness and induration [23]. MPCV-A is easier to induce fever than MPCV-AC. Seven cases of anaphylactic shock linked to AEFI were reported from 2015 to 2018 with an incidence of 0.006/100,000 doses, 2 of them were caused by immunization program vaccine (DTaP and MR), and 5 were rendered by PPV23 and rabies vaccine. Among AEFIs, anaphylactic shock is often caused by the residual ovalbumin or gelatin in vaccine gradients. The residual gradients in vaccine products were influenced by manufacture process. In China, the incidence of anaphylactic shock in AEFIs is between 0.005/100,000 doses − 0.08/100,000 doses. It is higher than that in USA with an incidence from 0.02/100,000 doses to 0.13/100,000 doses [24, 25]. This difference may be varied from the different process in various countries.

There are some limitations in the study. Firstly, the actual number of inoculants and the reported number of inoculants may not be completely consistent. In addition to obtaining the report of the number of inoculants from the system, we verified the actual number of inoculants from the aspects of birth population, floating population and vaccination rate to ensure the accuracy of the number of inoculants as far as possible; Secondly, differences in AEFI monitoring reports, disease diagnosis abilities and investigation abilities among different regions definitely exist. We do our best to ensure that all monitors are trained in standardization and can ensure the accuracy of the data.

## Conclusion

Although the incidence of AEFI in Jiangsu province increased from 2015 to 2018, common vaccine reactions were predominant while the incidence rates of severe abnormal reactions such as anaphylactic shock and VAPP were very rare. The continuous improving sensitivity in monitoring AEFIs together with standardized training in community doctors may be useful to decrease the incidence of developing severe AEFIs.

## Data Availability

The datasets used in this study are available from the corresponding author on reasonable request.

## Abbreviations

VAPP: Vaccine-associated paralytic poliomyelitis
AEFI: Adverse events following immunization
AEFIIS: Adverse events following immunization information system
